# The Impact of the COVID-19 Pandemic on the Clinical Course of Influenza in Hospitalised Children in the Years 2017–2025

**DOI:** 10.3390/life16010154

**Published:** 2026-01-17

**Authors:** Zuzanna Wasielewska, Justyna Franczak, Krystyna Dobrowolska, Justyna Moppert, Małgorzata Sobolewska-Pilarczyk, Małgorzata Pawłowska

**Affiliations:** 1 Department of Infectious Diseases and Hepatology, Collegium Medicum in Bydgoszcz, Nicolaus Copernicus University, 87-100 Toruń, Poland; 2 Collegium Medicum, Jan Kochanowski University, 25-369 Kielce, Poland

**Keywords:** clinical course, COVID-19, influenza, child

## Abstract

Background: The COVID-19 pandemic substantially altered the epidemiology of respiratory infections. Its impact on the clinical course of influenza in hospitalised children remains insufficiently characterised. Objectives: We aimed to compare the clinical course, complications, and selected laboratory parameters of influenza in children before, during, and after the COVID-19 pandemic. Methods: This single-centre retrospective study included 553 children hospitalised with laboratory-confirmed influenza between September 2017 and August 2025. Patients were divided into three groups: pre-pandemic, pandemic, and post-pandemic. Clinical complications and inflammatory markers (CRP, PCT, neutrophil counts) were analysed. Results: Influenza-related complications occurred in 59.5% of patients and were significantly more frequent after the pandemic compared to the pre-pandemic period (64.3% vs. 52.9%, *p* = 0.02). Pneumonia was the most common complication across all groups, but its incidence was lowest during the pandemic. Myositis occurred most frequently during the pandemic and appears to coincide with a higher proportion of influenza B infections. No significant differences were observed in CRP, PCT concentrations, or neutropenia rates between groups. Conclusions: The COVID-19 pandemic influenced the clinical presentation of influenza in children, with a post-pandemic increase in complications. These findings may reflect delayed access to healthcare and the phenomenon of immunity debt, highlighting the need for continued surveillance and preventive strategies.

## 1. Introduction

Influenza is an infectious disease caused by the influenza virus and affects both the paediatric and adult populations. Seasonal epidemic infections are primarily caused by influenza viruses of type A (approximately 80% of cases) and type B [1]. During the winter season in the United States, approximately 10% of hospitalisations in the paediatric population are associated with influenza, representing a significant financial burden on the healthcare system [2], similar to the high direct medical and societal costs in Poland [3]. Owing to airborne transmission via aerosol as well as direct contact, infections among children commonly occur in childcare and educational settings, such as nurseries, kindergartens, and schools [2].

The clinical presentation of influenza is dominated by respiratory tract symptoms (rhinorrhoea, cough, and sore throat), accompanied by fever and weakness or lethargy (accounting for approximately 85% of symptoms). Less commonly, gastrointestinal symptoms (vomiting, loose stools, and abdominal pain) or musculoskeletal pain may occur. Children under 5 years of age—particularly those under 2 years, including preterm infants—as well as children with chronic diseases of the heart, kidneys, lungs, or liver, haematological, metabolic, or oncological conditions, and those with congenital or acquired immunodeficiency, are at increased risk of a severe disease course and complications [1,2].

Bacterial infections of the respiratory tract (including pneumonia, bronchitis, otitis media, laryngitis, and pharyngitis), febrile seizures, and myositis constitute the most common complications of influenza in the paediatric population [2]. Diagnostic evaluation most frequently relies on widely available antigen tests, with molecular testing using real-time polymerase chain reaction (RT-PCR) employed less often [2,4].

Aetiological treatment for influenza is available, and its effectiveness increases the earlier the medication is administered following the onset of initial symptoms [2]. According to the American Academy of Pediatrics (AAP), aetiological treatment should be initiated in all children hospitalised with confirmed or suspected influenza, in those with influenza-related complications, in cases where there is a lack of response to symptomatic treatment, and in all children under 5 years of age and in children belonging to groups at increased risk of influenza complications. Oseltamivir (widely available in Poland) is the preferred antiviral agent owing to its safety profile, ease of administration, cost, and many years of clinical experience with its use [5].

Annual influenza vaccination is an effective and safe method of preventing influenza infection and reduces the risk of complications [1].

On 7 January 2020, a novel virus, SARS-CoV-2, was identified as the aetiological agent of the disease later named COVID-19 [6]. The rapid and efficient transmission of the virus led to a global pandemic within a short period of time, which reached Poland in March 2020. A state of epidemic was declared in Poland (formally lifted in May 2022), resulting in numerous changes in the organisation of education, healthcare services, and movement in public spaces [7,8,9]. On 5 May 2023, the World Health Organization (WHO) announced that COVID-19 no longer constituted a Public Health Emergency of International Concern (PHEIC), marking the end of the emergency phase of the pandemic rather than the complete cessation of global SARS-CoV-2 circulation [10].

In the broadly defined field of public health, the pandemic influenced the epidemiology and clinical presentation of many diseases. It appears that it may have also affected the clinical course and complications of influenza among children.

The aim of the present study was to compare the clinical course and complications of influenza in the paediatric population before, during, and after the COVID-19 pandemic era, corresponding to periods of hospital admission availability, based on a cohort of children hospitalised in the Department of Paediatrics, Infectious Diseases and Hepatology between 2017 and 2025.

## 2. Materials and Methods

The data were collected between 1 September 2017 and 31 August 2025 at a single paediatric infectious disease centre. The study population included 553 paediatric patients, all of whom were treated at the Department of Paediatrics, Infectious Diseases and Hepatology for influenza virus infection (International Classification of Diseases, ICD-10 codes J10.0, J10.1 and J10.8). The ICD-10 diagnosis was established on the basis of a positive antigen or molecular test confirming influenza A or B virus infection.

The analysed data included laboratory test results, including C-reactive protein (CRP) and procalcitonin (PCT) concentrations, as well as minimum and maximum neutrophil counts. Neutropenia was classified as mild (1.1–1.49 × 10^3^/µL), moderate (0.5–1.0 × 10^3^/µL), or severe (<0.5 × 10^3^/µL) based on the lowest neutrophil count recorded during hospitalisation, using fixed absolute thresholds applied uniformly across all age groups. The number and type of influenza-related complications were also analysed. Pneumonia was diagnosed based on radiological findings or, when imaging was not performed, on consistent clinical and auscultatory findings documented by the attending physician, reflecting real-world clinical practice across the study period. Myositis was diagnosed on the basis of elevated creatine phosphokinase (CPK) activity and reported muscle or limb pain and/or observed gait disturbances. Elevated creatine phosphokinase (CPK) activity was defined as values exceeding age-adjusted upper reference limits.

Three groups were distinguished according to specific time periods:

Group A—before the onset of the COVID-19 pandemic, i.e., from September 2017 to March 2020;

Group B—pandemic period, defined as October 2021 to May 2023. Although the COVID-19 pandemic was declared in March 2020, between April 2020 and September 2021, the study centre was dedicated exclusively to the hospitalisation of patients with COVID-19 and did not admit children with influenza. Therefore, to avoid misclassification and temporal dilution, Group B represents the period of resumed non-COVID admissions during the pandemic era rather than the entire pandemic timeline.

Group C—after the COVID-19 pandemic, i.e., from June 2023 to August 2025.

Patients were divided into age groups as follows:

- From the neonatal period to <2 years of age;
- From ≥2 years to <6 years of age;
- From 7 to 18 years of age.

During hospitalisation, all patients received antiviral therapy with oseltamivir, which was prescribed either as a newly initiated treatment or as a continuation of outpatient therapy, as well as supportive treatment in the form of fluid therapy and symptomatic management as indicated. Patients with acid–base or electrolyte disturbances received appropriate supplementation.

None of the hospitalised patients had been vaccinated against influenza during the infectious season in which they were hospitalised.

### 2.1. Ethics 

Patients were not exposed to any experimental interventions, and all treatment was provided in accordance with current clinical guidelines [11]. Personal data protection regulations were observed; therefore, informed consent was not required.

### 2.2. Statistical Analysis

Categorical variables (age distribution, sex distribution, influenza virus type, and complications) were described using numbers and percentages. Group comparisons for categorical variables were performed using Pearson’s χ^2^ test. For continuous variables, the Shapiro–Wilk test was used to assess data distribution. As the dataset did not follow a Gaussian distribution, continuous variables were reported as medians with interquartile ranges (IQR). The non-parametric Mann–Whitney U test was used for pairwise comparisons between groups (A vs. B, A vs. C, and B vs. C). To account for multiple comparisons, the Bonferroni correction was applied.

Statistical significance was defined as a *p*-value < 0.05. All analyses were performed using Statistica v.13 (StatSoft, Tulsa, OK, USA).

## 3. Results

During the analysed period, 553 patients diagnosed with influenza were hospitalised in the Department of Paediatrics, Infectious Diseases and Hepatology. In Group A, 223 patients were hospitalised; in Group B, 120 patients; and in Group C, 210 patients. The youngest hospitalised child was admitted on the 14th day of life, while the oldest patient reached 18 years of age during hospitalisation. In all groups, influenza type A infections predominated overall. Table 1 presents the number of children in Groups A, B, and C, stratified by age group, sex, and aetiological factor (influenza virus type A or B).

Differences in sex distribution and influenza virus type across groups were analysed descriptively, as these variables were considered potential confounders rather than primary outcomes.

Among children hospitalised due to influenza, influenza-related complications were diagnosed in 329 of 553 patients (59.49%), including 118 of 223 children (52.91%) in Group A, 76 of 120 children (63.33%) in Group B, and 135 of 210 children (64.29%) in Group C. “Any complication” refers to the presence of at least one influenza-related complication per patient; individual patients could present with multiple complications simultaneously. Influenza-related complications were observed significantly more frequently in children hospitalised after the COVID-19 pandemic (Group C) than in those hospitalised before the pandemic (Group A) (*p* = 0.02; Table 2). Table 2 presents the types of influenza-related complications and their frequencies in Groups A, B, and C.

Pneumonia was most frequently diagnosed among children assigned to Group A, in whom it was identified in 98 patients, accounting for 43.95% of the study group. In Group C, pneumonia was diagnosed in 28.57% of children. The lowest frequency of pneumonia was observed in Group B, where the diagnosis was made in 20 patients (16.67%). Comparative analysis between the individual groups demonstrated statistically significant differences in the incidence of pneumonia, with statistical significance observed both in the comparison of Group A with Group B (*p* < 0.001) and Group A with Group C (*p* < 0.001). The difference between Groups B and C did not reach statistical significance.

Gastrointestinal symptoms occurred with a similar frequency in children hospitalised before and during the COVID-19 pandemic (Group A: 17.49%; Group B: 16.67%); however, an increase was observed after the pandemic, affecting 23.81% of children in Group C.

Bacterial upper respiratory tract infections were identified in 7 children (3.33%) in Group C and occurred significantly less frequently compared with children in Group A (21 children; 9.42%) and Group B (12 children; 10.0%). Statistically significant differences were demonstrated when comparing Groups A vs. C (*p* = 0.03) and B vs. C (*p* = 0.03).

Myositis was most frequently diagnosed in children hospitalised during the COVID-19 pandemic (13.33%), and a statistically significant difference was demonstrated when these data were compared with the number of diagnoses in Group A (*p* < 0.001). Myositis was least frequently observed in Group A (0.9%), and comparison with Group C (in which the diagnosis was established in 13 cases; 6.19%) also reached statistical significance (*p* = 0.009).

Systemic inflammatory response syndrome (SIRS) was diagnosed in 26 children (11.66%) in Group A, 11 children (9.17%) in Group B, and 14 children (6.67%) in Group C. Differences in the frequency of SIRS among the groups were not statistically significant. Although a numerical decrease in SIRS frequency was observed across the study periods, this trend did not reach statistical significance.

Pericarditis was diagnosed in three children (1.35%) hospitalised before the COVID-19 pandemic (Group A); no children with cardiologic complications were hospitalised in subsequent years.

Neutropenia—classified as mild (1.1–1.49 × 10^3^/µL), moderate (0.5–1.0 × 10^3^/µL), or severe (<0.5 × 10^3^/µL)—was diagnosed based on the lowest neutrophil count recorded during hospitalisation. These thresholds were applied uniformly to all patients in accordance with routine clinical practice at the study centre. Although age-adjusted reference ranges may provide greater physiological precision, retrospective reclassification according to age-specific norms was not feasible. Therefore, neutropenia was analysed comparatively between study groups rather than as an absolute prevalence estimate. Neutropenia was observed in 105 children (47.09%) in Group A, 46 children (38.33%) in Group B, and 101 children (48.1%) in Group C. Neutropenia occurred least frequently during the COVID-19 pandemic; however, no statistically significant differences were found when comparing all groups. Table 3 presents the analysed laboratory parameters in Groups A, B, and C.

The analysed median concentrations of laboratory inflammatory markers (CRP and PCT) were similar across all groups (A, B, and C), and no statistically significant differences were identified.

Among the hospitalised children, subgroups with elevated C-reactive protein (CRP >60 mg/dL) and procalcitonin (PCT >2 ng/mL) concentrations were identified. Elevated CRP levels were observed more frequently in children from Group B (7.5%) compared with Group C (3.81%) and Group A (4.52%); however, these differences were not statistically significant. Similar observations were made for elevated PCT concentrations, which were most frequently detected among children hospitalised during the COVID-19 pandemic (Group B: 8.41%), slightly less frequently before the pandemic (Group A: 7.6%), and least frequently in the post-pandemic period (Group C: 6.15%). The observed differences were not statistically significant.

**Figure 1:**
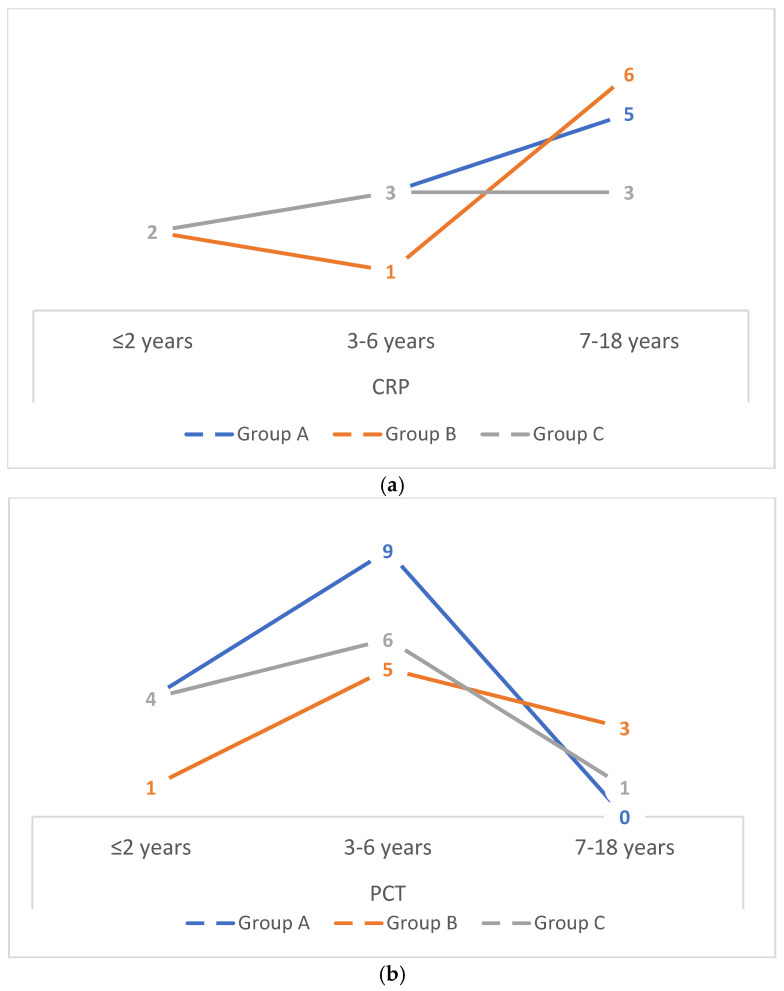
Age distribution of paediatric patients infected with the influenza virus with C-reactive protein levels >60 mg/dL (**a**) and procalcitonin levels >2 ng/mL (**b**) in Groups A, B, and C. Due to small subgroup sizes, these analyses are descriptive and intended to illustrate distribution patterns rather than infer statistical significance. Absolute numbers of patients are provided above each bar.

Figure 1 presents the number of hospitalised children with elevated inflammatory markers—CRP >60 mg/dL (a) and PCT >2 ng/mL (b)—stratified by age group in Groups A, B, and C. In all groups (A, B, and C), the highest number of children with elevated CRP concentrations was observed among those older than 7 years, whereas elevated PCT concentrations were most frequently detected in children aged 3–6 years.

## 4. Discussion

At present, there are very few publications in the literature analysing the clinical course of influenza in the paediatric population before, during, and after the COVID-19 pandemic; therefore, comparison of our results with observations reported by other authors is difficult or, in some aspects, impossible. Among children hospitalised for influenza in our department, complications were identified in all study groups: in 118 children (52.91%) in Group A, 76 children (63.33%) in Group B, and 135 children (64.29%) in Group C. An increase in the frequency of complications (by 10.42 percentage points) was already observed during the COVID-19 pandemic compared with the pre-pandemic period; however, statistical significance (*p* = 0.02) was demonstrated only in the comparison between Group A and Group C (i.e., before and after the pandemic).

No a priori sample size calculation was performed due to the retrospective nature of the study. Group sizes were determined by the number of hospitalised influenza cases during predefined time periods. Consequently, the study may be underpowered to detect small-to-moderate differences for selected outcomes, particularly in Group B. Non-significant findings should therefore be interpreted with caution and cannot be definitively regarded as evidence of absence of effect. Age-stratified analyses and multiple comparisons were interpreted descriptively, acknowledging an increased risk of type I and type II errors.

The frequency of influenza-related complications among children in the pre-pandemic period (Group A; 52.91%) is comparable to the findings reported by Wrotek et al., who demonstrated that influenza complications affected 57.3% of hospitalised children during the 2015/2016 infectious season [3].

Available literature includes studies indicating that during the first months of the COVID-19 pandemic, parents, fearing SARS-CoV-2 infection, presented to hospitals later, and children were observed to have a clinically more severe course of infection. In a survey study conducted among parents presenting with their children to hospital emergency departments or admission units during the initial months of the COVID-19 pandemic, Davis et al. demonstrated that nearly one in five caregivers sought hospital care for their ill child with a delay, with fear of contracting COVID-19 identified as the main reason for postponement. Among these children, 43.2% experienced a deterioration in health status resulting from delayed contact with healthcare services [11], which may have contributed to a higher number of complications.

Another possible explanation for the observed changes in complication frequency may be the phenomenon of so-called “immunity debt”, which has been proposed in the literature but remains speculative in the context of influenza [12,13].

The concept of immunity debt has been proposed as a potential explanatory framework for post-pandemic increases in respiratory infections; however, its applicability to influenza remains speculative. Influenza immunity differs from that of viruses such as RSV, and the present study lacks individual-level data on prior influenza exposure or immunological status. Therefore, references to immunity debt should be interpreted as hypothesis-generating rather than as evidence of a causal mechanism. Alternative explanations, including changes in healthcare-seeking behaviour, admission thresholds, clinical documentation practices, and viral evolution, must also be considered.

Similar observations emerge from our own study, in which the proportion of complications increased among children hospitalised during and after the pandemic (Groups B and C) compared with those hospitalised before the pandemic (Group A). The difference in complication rates between Groups A and C was statistically significant (*p* = 0.02).

Among influenza-related complications, pneumonia was the most frequently observed complication across all analysed groups. Similar observations were reported by Bennet et al. based on a study conducted in Sweden between 1998 and 2014. They demonstrated that complications occurred in 380 of 922 (41%) children with influenza, among whom pneumonia affected 117 children and was the most commonly diagnosed complication [14]. In Polish studies by Siewert et al. [15] and Wrotek et al. [3], pneumonia was also the most frequently observed complication, affecting 25.5% (Siewert) and 31% (Wrotek) of children, respectively. Stopyra et al., in a publication concerning the 2023/2024 influenza season, reported pneumonia in 20% of children with influenza, which corresponds with our observations regarding the frequency of pneumonia in Group C (28.57%) [16].

In a retrospective cohort study, Lin et al. analysed the clinical course and laboratory findings in 885 children with influenza A before, during, and after the COVID-19 pandemic and, similarly to our study, demonstrated that pneumonia was least frequently diagnosed during the COVID-19 pandemic period (corresponding to Group B in our study) [17]. Furthermore, Chiapinotto et al. showed in 2023 that during the COVID-19 pandemic, increasing restrictions and mandatory social distancing were associated with a significant reduction in hospitalisations due to lower respiratory tract infections among children [18]. These observations appear to be consistent with our data. Similar anticyclical patterns between respiratory virus circulation and SARS-CoV-2 activity have been described by Kiefer et al., who demonstrated viral interference between RSV hospitalisation waves and COVID-19 circulation in a paediatric population, providing mechanistic support for reduced respiratory complications during periods of intense pandemic control measures [19].

There are well-documented immunomodulatory mechanisms between the influenza virus and respiratory tract bacteria that promote bacterial colonisation [20], predisposing patients to secondary bacterial infections of the upper respiratory tract, which were also observed in our patient population.

In the previously cited analysis by Lin et al., an almost identical frequency of gastrointestinal symptoms was reported in children in the pre-pandemic period (15.06% in the cited study) compared with Group A in the present study (17.49%). Similarly to our findings, the highest proportion of children with vomiting and loose stools was observed after the COVID-19 pandemic (Group C) [17]. In both studies, myositis was least frequently diagnosed in the pre-pandemic period [17]. However, analysis of our data revealed that the higher the proportion of influenza B infections within a given group (A, B, or C), the more frequently myositis was diagnosed among hospitalised children. Agyeman et al., in a study of 316 patients with influenza-associated myositis, reported that influenza virus type B was the aetiological agent in 76% of cases [21]. The increased frequency of myositis observed in children hospitalised during the COVID-19 pandemic coincided with a substantially higher proportion of influenza B infections in this period. Previous studies have demonstrated a strong association between the influenza B virus and influenza-associated myositis. These observations are further supported by the findings of Şık et al., who identified distinct clinical phenotypes of severe paediatric influenza in the post-COVID period, with influenza B infections disproportionately associated with neurological and musculoskeletal complications [22]. Therefore, the observed increase in myositis is more likely attributable to virus type rather than to the pandemic period itself. However, due to the retrospective design and limited subgroup sizes, residual confounding by age or virus type cannot be excluded.

A critical confounding factor in the interpretation of temporal trends is the substantial shift in influenza virus type distribution across the study periods. Group B was characterised by a markedly higher proportion of influenza B infections compared with the pre-pandemic and post-pandemic periods. Influenza B has been previously associated with a higher risk of musculoskeletal and neurological complications, including myositis. Therefore, differences in complication profiles—such as increased myositis and lower pneumonia rates observed during the pandemic period—may be partially or predominantly attributable to viral subtype rather than pandemic-related factors alone. Due to limited subgroup sizes, stratified or multivariable analyses adjusting for virus type were not feasible, and this represents an important limitation of causal inference.

In the literature, systemic inflammatory response syndrome (SIRS) is described as a non-specific inflammatory response that may occur in both bacterial and viral infections, including influenza, and is rarely observed in children. Our observations indicate that the frequency of SIRS was relatively high among hospitalised children and did not differ significantly between the groups (6.67–11.66%). Children with influenza most often require hospitalisation in cases of a complicated disease course; in Europe, this concerns only 1–3% of paediatric patients. These findings are consistent with earlier reports suggesting that severe systemic inflammatory responses during viral infections in children are uncommon [23,24].

Reports on cardiological complications such as pericarditis or myocarditis in the course of influenza in children are based mainly on individual case reports and small case series, reflecting the rarity of these conditions [25]. The few available analyses concern adult populations, with conclusions suggesting that pericarditis occurs more frequently in men and older individuals and is more often associated with influenza type A infection [26].

Analysis of laboratory inflammatory markers showed that CRP and PCT concentrations, as well as neutrophil counts, were characteristic of a viral infection such as influenza. In our analysis, the median CRP concentration ranged from 6.25 to 6.3 mg/dL; although these values were slightly above the upper limit of normal (N <5 mg/dL), they were indicative of a viral rather than a bacterial infection. Findings similar to ours were reported in a retrospective study of 36,047 children hospitalised for influenza in the pre-pandemic period (June 2014–June 2019), in which Shi et al. demonstrated that among children with a mild disease course, the mean CRP concentration was 8 mg/dL (median 0.6–8 mg/dL) [27]. Interestingly, in our study, the number of children with elevated CRP and PCT concentrations did not overlap when the occurrence of these markers was analysed across the individual age groups.

The interpretation of inflammatory markers requires caution. Although median CRP and PCT values did not differ significantly between groups, wide interquartile ranges indicate substantial within-group variability. The thresholds used for CRP (>60 mg/dL) and PCT (>2 ng/mL) were selected as markers of potential severe inflammation based on clinical practice rather than validated prognostic cut-offs for bacterial co-infection in influenza. Data on microbiologically confirmed bacterial co-infections and antibiotic prescribing patterns were not uniformly available and could not be reliably analysed. Consequently, dichotomised analyses should be regarded as descriptive rather than diagnostic.

Neutropenia was observed in a substantial proportion of children, occurring in 47.09% of patients in Group A, 38.33% during the pandemic period, and 48.1% in the post-pandemic period; these differences were not statistically significant. In contrast, a markedly lower proportion of neutropenia was reported among hospitalised Turkish children (23.7%); however, no information was provided regarding the neutrophil count thresholds used for the diagnosis of neutropenia, and the authors did not perform an analysis stratified by the severity of neutropenia [28].

An important observation of this study is that none of the hospitalised children had received influenza vaccination during the season of infection. This finding should be interpreted with caution, as influenza vaccination coverage among children in Poland remains low. In addition, vaccination status was obtained retrospectively from medical records and may be subject to incomplete documentation. The absence of vaccinated children among hospitalised patients limits external validity and precludes any conclusions regarding vaccine effectiveness.

From a public health perspective, a systematic review by Raycheva et al. demonstrated that combined COVID-19 control strategies—including vaccination, testing, and social distancing—were the most cost-effective approaches, providing important context for the broader preventive impact of pandemic-related measures on respiratory infection burden [29].

### Limitations

This study has several limitations. First, its retrospective, single-centre design limits generalizability and is subject to incomplete documentation. Second, the exclusion of outpatient and emergency department-managed influenza cases may bias the cohort toward more severe disease presentations. Third, the temporary reorganisation of the hospital during the COVID-19 pandemic resulted in a condensed pandemic-period cohort, introducing temporal discontinuity and potential selection bias. Fourth, the lack of viral sequencing or viral load data precludes assessment of variant-related severity differences. Fifth, data on timing of antiviral therapy initiation were not uniformly available and could not be analysed. Finally, the observational design does not allow causal inference regarding pandemic-related effects, immunity debt, or healthcare system changes.

In particular, the condensed pandemic-period cohort should be interpreted as representative of influenza cases hospitalised during periods of resumed non-COVID admissions rather than the entire pandemic timeline.

## 5. Conclusions

The COVID-19 pandemic and its aftermath were associated with observed differences in the frequency and pattern of influenza-related complications in hospitalised children. Pneumonia remained the most common complication, with the lowest incidence observed during the pandemic period.

Myositis occurred more frequently during the pandemic and coincided with a higher proportion of influenza B infections, suggesting a virus-type effect rather than a direct pandemic-related influence.

No significant differences were observed in inflammatory markers or neutropenia rates across the analysed periods. These findings should be interpreted as descriptive and hypothesis-generating, highlighting the need for further multicentre studies.

## Figures and Tables

**Table 1:** Characteristics of the study population according to age, sex, and aetiological factors (influenza virus type A or B) in Groups A, B, and C.

Parameter	Group A	Group B	Group C
Number of children	223	120	210
Sex, girls/boys, *n* (%)	87 (39.01)/136 (60.99)	58 (48.33)/62 (51.67)	107 (50.95)/103 (49.05)
Influenza virus type A/B, *n* (%)	199 (89.24)/24 (10.76)	68 (56.67)/52 (43.33)	159 (75.71)/51 (24.29)
Age distribution, *n* (%)			
≤2 years	61 (27.35)	24 (20)	65 (30.95)
3–6 years	80 (35.87)	38 (32.50)	67 (31.90)
7–18 years	82 (36.77)	58 (47.50)	78 (37.14)

**Table 2:** Influenza-related complications in hospitalised paediatric patients in Groups A, B, and C.

Parameter	Group A *n* = 223	Group B *n* = 120	A vs. B *p*-Value	Group C *n* = 210	A vs. C *p*-Value	B vs. C *p*-Value
Any complication, *n* (%)	118 (52.91)	76 (63.33)	0.07	135 (64.29)	0.02	0.91
SIRS, *n* (%)	26 (11.66)	11 (9.17)	0.99	14 (6.67)	0.20	0.99
Gastrointestinal symptoms, *n* (%)	39 (17.49)	20 (16.67)	0.99	50 (23.81)	0.30	0.39
Pneumonia, *n* (%)	98 (43.95)	20 (16.67)	<0.001	60 (28.57)	<0.001	0.06
Bacterial upper respiratory tract infection, *n* (%)	21 (9.42)	12 (10)	0.99	7 (3.33)	0.03	0.03
Skeletal muscle inflammation, *n* (%)	2 (0.90)	16 (13.33)	<0.001	13 (6.19)	0.009	0.99
Pericarditis, *n* (%)	3 (1.35)	0	0.60	0	0.27	n/a

Abbreviations: SIRS—Systemic Inflammatory Response Syndrome.

**Table 3:** Comparison of inflammatory marker levels (CRP, PCT) and neutrophil counts in hospitalised children in Groups A, B, and C.

Parameter	Group A (*n* = 223)	Group B (*n* = 120)	Group C (*n* = 210)	*p*-Value A vs. B	*p*-Value A vs. C	*p*-Value B vs. C
Neutropenia, *n* (%)						
Mild (1.1–1.49 × 10^3^/µL)	26 (11.66)	14 (11.67)	34 (16.19)	0.99	0.60	0.99
Moderate (0.5–1.0 × 10^3^/µL)	38 (17.04)	20 (16.67)	44 (20.95)	0.99	0.60	0.99
Severe (<0.5 × 10^3^/µL)	41 (18.39)	12 (10.00)	23 (10.95)	0.27	0.27	0.99
CRP, mg/dL, median (IQR)	6.3 (1.6–20.6)	6.3 (1.55–22.8)	6.25 (1.45–19.45)	0.44	0.92	0.49
PCT, ng/mL, median (IQR)	0.14 (0.07–0.57)	0.19 (0.10–0.47)	0.16 (0.08–0.45)	0.09	0.55	0.19
CRP > 60 mg/dL, *n* (%)	10 (4.52) *n* = 221	9 (7.50)	8 (3.81)	0.32	0.81	0.19
PCT > 2 ng/mL, *n* (%)	13 (7.60) *n* = 171	9 (8.41) *n* = 107	11 (6.15) *n* = 179	0.82	0.67	0.48

## Data Availability

The original contributions presented in this study are included in the article. Further inquiries can be directed to the corresponding author.

## Abbreviations

The following abbreviations are used in this manuscript:

WHO	World Health Organization
IQR	Interquartile Range
COVID-19	Coronavirus Disease 2019
AAP	American Academy of Pediatrics
CI	Confidence Interval
SARS-CoV-2	Severe Acute Respiratory Syndrome Coronavirus 2
PCR	Polymerase Chain Reaction
ICD-10	International Classification of Diseases
CRP	C-reactive Protein
PCT	Procalcitonin
CPK	Creatine Phosphokinase
SIRS	Systematic Inflammatory Response Syndrome
